# Combined Transcriptome and Proteome Leukocyte’s Profiling Reveals Up-Regulated Module of Genes/Proteins Related to Low Density Neutrophils and Impaired Transcription and Translation Processes in Clinical Sepsis

**DOI:** 10.3389/fimmu.2021.744799

**Published:** 2021-09-10

**Authors:** Giuseppe Gianini Figueirêdo Leite, Bianca Lima Ferreira, Alexandre Keiji Tashima, Erika Sayuri Nishiduka, Edecio Cunha-Neto, Milena Karina Colo Brunialti, Murillo Assuncao, Luciano Cesar Pontes Azevedo, Flávio Freitas, Tom van der Poll, Brendon P. Scicluna, Reinaldo Salomão

**Affiliations:** ^1^Division of Infectious Diseases, Department of Medicine, Escola Paulista de Medicina, Universidade Federal de Sao Paulo, São Paulo, Brazil; ^2^Department of Biochemistry, Escola Paulista de Medicina, Universidade Federal de Sao Paulo, São Paulo, Brazil; ^3^Laboratorio de Imunologia, Instituto do Coracao, Hospital das Clinicas da Faculdade de Medicina da Universidade de Sao Paulo, São Paulo, Brazil; ^4^Intensive Care Unit, Hospital Israelita Albert Einstein, São Paulo, Brazil; ^5^Intensive Care Unit, Hospital Sirio Libanes, São Paulo, Brazil; ^6^Intensive Care Unit, Hospital São Paulo, Escola Paulista de Medicina, Universidade Federal de São Paulo, São Paulo, Brazil; ^7^Center for Experimental and Molecular Medicine, Amsterdam University Medical Centers, Academic Medical Center, University of Amsterdam, Amsterdam, Netherlands; ^8^Division of Infectious Diseases, Amsterdam University Medical Centers, Academic Medical Center, University of Amsterdam, Amsterdam, Netherlands; ^9^Department of Applied Biomedical Sciences, Faculty of Health Sciences, Mater Dei hospital, University of Malta, Msida, Malta

**Keywords:** LDNs, omics, transcriptional shutdown, translation shutdown, protein-protein interaction

## Abstract

Sepsis is a global health emergency, which is caused by various sources of infection that lead to changes in gene expression, protein-coding, and metabolism. Advancements in “omics” technologies have provided valuable tools to unravel the mechanisms involved in the pathogenesis of this disease. In this study, we performed shotgun mass spectrometry in peripheral blood mononuclear cells (PBMC) from septic patients (N=24) and healthy controls (N=9) and combined these results with two public microarray leukocytes datasets. Through combination of transcriptome and proteome profiling, we identified 170 co‐differentially expressed genes/proteins. Among these, 122 genes/proteins displayed the same expression trend. Ingenuity Pathway Analysis revealed pathways related to lymphocyte functions with decreased status, and defense processes that were predicted to be strongly increased. Protein-protein interaction network analyses revealed two densely connected regions, which mainly included down‐regulated genes/proteins that were related to the transcription of RNA, translation of proteins, and mitochondrial translation. Additionally, we identified one module comprising of up‐regulated genes/proteins, which were mainly related to low-density neutrophils (LDNs). LDNs were reported in sepsis and in COVID-19. Changes in gene expression level were validated using quantitative real-time PCR in PBMCs from patients with sepsis. To further support that the source of the upregulated module of genes/proteins found in our results were derived from LDNs, we identified an increase of this population by flow cytometry in PBMC samples obtained from the same cohort of septic patients included in the proteomic analysis. This study provides new insights into a reprioritization of biological functions in response to sepsis that involved a transcriptional and translational shutdown of genes/proteins, with exception of a set of genes/proteins related to LDNs and host‐defense system.

## 1 Introduction

In the past few decades, sepsis has gained immense attention as a global health emergency that affects millions of people worldwide. Clinically, it is defined as a life‐threatening organ dysfunction caused by the dysregulated host response to infection (1). Despite the advancements in the diagnosis and treatment strategies, high prevalence and mortality rates of sepsis have been recorded over the last 20 years (2). Additionally, the high cost of treatment associated with sepsis makes it one of the most expensive healthcare problems.

The complex pathophysiology of sepsis is largely dependent on the host’s immune response against microbial infections (3, 4). The patients’ outcome is determined by fine regulation of a variety of genes, proteins, metabolites, and other molecules, which are associated with different pathways that play a pivotal role in disease pathogenesis (4–6). In particular, a fine balance between the molecules involved in inflammatory and anti‐inflammatory responses increases the likelihood of infection control and organ function recovery. However, the predominance of either of the two responses might result in poor disease outcomes, which could be attributed to organ dysfunction and persistence of primary infection or breakthrough of secondary infections (4–6).

In the last decades, the development and advancement of “omics” technologies revolutionized the field of sepsis research. These technologies ensured a better understanding of disease pathogenesis (7, 8) and enabled the discovery and validation of important disease biomarkers (9). The application of transcriptomics to whole blood leukocytes provided important insights into the pathophysiology of sepsis. In particular, these studies mediated the identification of sepsis genomic signatures (9, 10) and provided useful information regarding the alteration of associated biological pathways, which further enabled the discovery of potential therapeutic strategies (11, 12). In addition to this, the analyses and conclusions drawn from these initial studies encouraged the in‐depth search to unravel any hidden information present in the high‐dimensional transcriptomic data (13). Proteomics studies previously conducted for sepsis provided valuable information regarding changes in various biological pathways, including lipid metabolism, cytoskeleton and cellular assembly (7, 14). In some of the studies, the application of protein-protein interaction networks (PPINs) and transcriptome/proteome profiling enabled the identification of biomarkers, disease‐related pathways, and proteins associated with patients’ survival (7, 15–17).

In the present study, concepts of network biology were employed to evaluate the systemic host response in patients with sepsis. The study aimed to utilize PPIN analysis to gain better insights into the interconnections present between the genes/proteins with highly altered expression, and thus unravel the functional modules involved in the biological processes associated with clinical sepsis. For this purpose, we performed shotgun mass spectrometry in a cohort of septic patients combined with two publicly microarray dataset to identify co-differentially expressed genes/proteins and overrepresented pathways. Results were validated by qPCR and flow-cytometry.

## 2 Materials and Methods

### 2.1 Transcriptome Analysis

#### 2.1.1 Collection of Microarray Data

Two publicly available microarray datasets GSE65682 (SD1) and E-MTAB-5273 (SD2) were obtained from the Gene Expression Omnibus and Arrayexpress, respectively. These datasets were selected from other datasets with reference to the following criteria: i) dataset providing data information about gender and age; ii) the presence of corresponding controls in the same dataset (healthy individuals or individuals scheduled for elective surgery); iii) available processed data and iv) samples collected within 24 h after ICU admission. The exclusion criteria include: i) animal and pediatric studies and ii) datasets with small number of samples (**Table 1** and **Supplementary Material 1**).

**Table 1:** Datasets information.

Access	# Patients: Controls	Samples	Reference
GSE65682 (SD1)	478:42	Whole blood	Scicluna, van Vught (9)
E-MTAB-5273 (SD2)	46:10	Blood leukocytes	Burnham, Davenport (11)

#### 2.1.2 Microarray Data Analysis

We read the processed data into the R environment (v. 4.0.2). First, the transcripts were collapsed to unique genes by calculating the mean expression of transcripts from the same gene locus using tidyverse and dplyr packages. The annotation file used to map the probes to genes was previously published in Khan, Perlee (18). Comparisons were performed using limma with Benjamini-Hochberg (BH) adjusted *p-value* < 0.05 and Log_2_ Fold-Change (FC) < −0.26 or > 0.26 significance thresholds.

#### 2.1.3 Venn Diagram Analysis of Differentially Expressed Genes

The InteractiVenn tool (http://www.interactivenn.net/) was used to overlap the lists of differentially expressed genes (DEGs) from the two different datasets. Then, a set of DEGs was selected based on those overlapping and following the same direction of expression (up-regulated or down-regulated). In addition, we selected only genes classified as “protein-coding” for further analysis.

### 2.2 Proteome Analysis

#### 2.2.1 Study Design and Sample Collection

Blood samples were collected from healthy volunteers and from patients with a clinical diagnosis of severe sepsis and septic shock, who were admitted to the intensive care units of Sao Paulo Hospital, Albert Einstein Hospital, and Sirio-Libanes Hospital during 2014–2016. The prospective study was approved by the ethics committees of the participating hospitals, and written informed consent was obtained from all participants or relatives before blood sampling. The diagnosis was made according to the ACCP/SCCM consensus conference (19) and then later adjusted to the revised concepts of sepsis and septic shock (1). The samples were obtained within 48 h of the first organ dysfunction or shock and stored in the biobank BR047 (CEP/UNIFESP-HSP). Patients aged <18 years, who were participating in any experimental treatment, undergoing chemotherapy, or in imminent death were excluded. After approval by the institutional ethics committee (CEP/UNIFESP 1171/2017), we selected 24 patients with sepsis secondary to community-acquired infections among the 134 patients enrolled in the cohort (**Table S1** in **Supplementary Material 1**) and 9 healthy age- and gender-matched volunteers for the proteomic analysis. For the gene expression analysis by qPCR, we selected seven patients and seven healthy volunteers.

#### 2.2.2 Preparation of PBMCs and Protein Sample Preparation

PBMC were isolated from blood samples using the Ficoll gradient method (Ficoll-Paque PLUS; GE Healthcare Bio-Sciences, Sweden) and stored in liquid nitrogen before use. After thawing, the protein extracts were obtained by lysis in 7 M urea, 2 M Thiourea, and 200 mM Dithiothreitol (DTT) with the Protease Inhibitor Cocktail (Sigma, USA). After centrifugation at 13,000 *g* for 15 min at 4°C, the protein concentration in the supernatants was determined using the Bradford method (20). The samples were reduced with 5 mM DTT at 65°C for 30 min and then alkylated with 15 mM iodoacetamide at room temperature for 30 min in the dark. The proteins were precipitated in acetone/methanol (8:1, v:v) at −80°C for 16 h and, after two washes with methanol, recovered by centrifugation at 14,000 *g* for 10 min at 4°C. They were then dissolved in 100 mM NaOH (5 µL/100 µg protein) and the volume was adjusted with 50 mM HEPES (pH 7.5) to a protein concentration of 1 µg.µL^-1^. The proteins were digested with trypsin (Promega, USA) at a 1:100 enzyme:protein ratio at 37°C overnight, and the digestion was stopped by the addition of 10 µL of 5% trifluoroacetic acid. The samples were then desalted using C18 tips, vacuum-dried, and stored at −80°C. Before the analysis, the peptides were suspended at 0.1% formic acid to a final concentration of 2 µg.µL^-1^.

#### 2.2.3 Mass Spectrometry Acquisition (LC-MS/MS)

LC-MS/MS analyses were performed in the chromatographic system nanoAcquity ultra performance liquid chromatography (UPLC) (Waters) coupled to the Synapt G2 HDMS Mass Spectrometer (Waters). Samples (10 µg) were loaded into a trap column (Acquity UPLC M-Class Symmetry C18 Trap Column,100 Å, 5 μm, 300 μm × 25 mm; Waters) at 8 μL/min of phase A (deionized water, 0.1% formic acid) for 5 min. Then, the mixture of trapped peptides was eluted in an analytical column (Acquity UPLC M-Class HSS T3 Column, 1.8 μm, 300 μm × 150 mm; Waters) with a gradient of 7%–35% of Phase B (0.1% formic acid in acetonitrile) over 60 min at a flow rate of 3 µL/min. The MS data were acquired on data-independent mode (DIA) using ion mobility separation UDMS^E^ (21). in the *m*/*z* range of 50–2000 and set up in the resolution mode. Peptide ions were fragmented by collision-induced dissociation (CID), in which collision energies were alternated between low (4 eV) and high (ramped from 17 to 60 eV) for precursor ion and fragment ions, respectively, using the scan time of 1.0 s. The ESI source was operated in the positive mode with a capillary voltage of 3.0 kV, block temperature of 100°C, and cone voltage of 40 V. The column temperature was set at 55°C, and the samples were maintained in an autosampler at 10°C. For lock mass correction, a [Glu1]-Fibrinopeptide B solution (500 fmol/mL in 50% acetonitrile, 0.1% formic acid; Peptide 2.0) was infused through the reference sprayer at 2 μL/min and sampled every 60 s for external calibration (22).

#### 2.2.4 Progenesis QI for Label-Free Quantification

Label-free quantification was performed in Progenesis QI for proteomics (NonLinear Dynamics, Newcastle, UK) as previously reported (23, 24). Briefly, the raw files were loaded in the software, and the samples were aligned based on the precursor ion retention time of the reference run, which was automatically selected. The default peak picking parameters were then applied. Searches for protein identification were run against *Homo sapiens* sequences from the UniprotKB/Swissprot (www.uniprot.org; reviewed; 26,426 sequences; downloaded on November 30, 2018). The database was combined with a list of 259 most common contaminants as well as examined with all their reverse sequences.

Search parameters were set as follows: carbamidomethyl cysteine residues were selected as fixed modification and N-terminal Acetylation and methionine oxidation as variable modifications. Ion account was selected as the peptide identification method with the search tolerance parameters set as automatic for peptide and fragment tolerance. The minimum ion match requirements for protein identifications were set to at least one fragment per peptide, five fragments per protein, and two peptides per protein. Up to two missed cleavage sites were allowed for trypsin digestion. A maximum peptide false discovery rate (FDR) of 1% was set at the peptide level. Proteins were quantified by the average signal intensity of the three most intense tryptic peptides of each protein (25).

#### 2.2.5 Bioinformatics Analysis

Progenesis output tables were previously filtered by removing reversed and contaminants hits and keeping only proteins with ≥2 peptides matched (*one of which was unique*). The resulting table was then imported and analyzed using the Perseus software (v. 1.6.15.0) (26). Briefly, data were log2 transformed, biological replicates were grouped, and then mass spectrometry intensity values were filtered to have at least 60% quantified values in either of the group (Sepsis or Healthy). Student’s t-test analyses were performed, and the Benjamini-Hochberg corrections was applied for all p-values to calculate the false discovery rates (FDR). For the selection of differentially expressed proteins (DEPs), we considered the following criteria*: p-value* < 0.05 and Log_2_FC < −0.37 or > 0.37 (27, 28).

### 2.3 Pathway Enrichment Analysis

We performed the canonical pathway enrichment analysis in the ingenuity pathway analysis (IPA, Qiagen Bioinformatics, ThermoFisher) in DEGs and DEPs separately to determine the most significantly affected pathways and the predicted activation state. To gain additional insights, we performed the diseases and functions analyses in IPA that displayed the predicted diseases or biological functions. The statistical significance *p*-value was calculated using Fischer’s exact test and adjusted for multiple comparisons by BH-adjusted *p*-values. We used the *z*-score >2 (pathway increased) or *z*-score <−2 (pathway decreased) with a B–H *p*-value <0.05 as the significance cutoff for our analysis. Then, we performed the comparative analysis in order to compare the similarity and difference among the enriched pathways and diseases or functions resulting from genes/proteins within each dataset.

### 2.4 Comparison and Correlations Between Transcriptome and Proteome Data

We analyzed the list of DEGs and DEPs looking for co-differentially expressed genes/proteins in the two lists. First, we converted the gene symbol to Entrez Gene IDs using the DAVID mapping service (https://david.ncifcrf.gov/conversion.jsp) and then and secondly, employed the InteractiVenn tool (http://www.interactivenn.net/) to uncover overlapping genes and protein products. Throughout this analysis we matched for directionality of expression patterns. Based in Shapiro–Wilk test we calculated the Spearman’s rank correlation coefficient as a measure of the strength of the relationship between messenger RNAs (mRNAs) and protein expression levels. For this purpose, we used the R environment (v. 4.0.2) for statistical was done by ggpubr (v. 0.4.0) and visualization by ggplot2 (v. 3.3.3).

### 2.5 Construction and Visualization of PPIN

The construction of the sepsis PPIN by co-differentially expressed genes/proteins were generated based on the interactions derived from the STRING database using the Cytoscape stringApp (v. 1.5.1) for the *H. sapiens* with a confidence cutoff score set to 0.7 and no additional interactors. The network was visualized in Cytoscape (v. 3.8.2), and the representation of the log2FC variation was created using the Omics Visualizer app (v. 1.3.0) (29). To group the proteins in the network based on their interactions from STRING (modules), we used the clusterMaker2 to run Markov clustering (MCL), with the following criteria: value to 4.0, set array sources to use the STRING confidence score attribute as weights and left all other settings at their default. We thus selected the largest cluster (module) in the network and performed functional enrichment analysis using the STRING enrichment API (FDR-corrected p-value <0.05) (30).

In order to expand the information from the modules, we created a global PPIN using all DEGs and DEPs, with the same interaction criteria as described above. In this case, we performed a multiscale community analysis to group the proteins. We used the “CyCommunityDetection” app. with the following criteria: Algorithm: OSLOM, weight column: STRING score, random number seed: 1, and all other settings at default. Then, we performed functional enrichment on the hierarchy network using g: Profiler as the algorithm (31).

### 2.6 Gene Expression Analysis by qPCR

Total RNA was isolated from PBMC using the RNEasy Mini-Kit (Qiagen, Germany), treated with DNAse I (Qiagen), and quantified in Nanodrop (Thermo Fisher Scientific, USA). Next, 500 ng of total RNA per sample was used for cDNA synthesis using the High-Capacity cDNA Reverse Transcription Kit with RNase Inhibitor (Thermo Fisher Scientific, Lithuania). The real-time polymerase chain reaction was performed in the 7500 Real-Time PCR System (Applied Biosystems, USA) with the gene-specific primers listed in **Table S2** in **Supplementary Material 1**. The relative gene expression was analyzed using the 2^-∆∆^ *^Ct^* method. The syntaxin-5 (*STX5*) gene was used as endogenous control (32, 33).

### 2.7 Immunophenotyping of Low-Density Neutrophils

PBMC from septic patients and healthy volunteers were thawed. Then, five hundred thousand cells were transferred to polystyrene tubes and stained with CD66b FITC clone G10F5 (BD Biosciences), CD45 BV421 clone HI30 (BD Biosciences) and CD16 PE clone 3G8 (BD Biosciences); 7-Amino-Actinomycin D (7-AAD; BD Biosciences) was included to assess cell viability. Samples were incubated for 15 min in the dark at room temperature. After washing, the cells were suspended in a buffer solution (PBS, 0.1% BSA, 2 mM EDTA) and analyzed by flow cytometry using a LSR FORTESSA (BD Biosciences). Data analysis was performed in the FlowJo software (FlowJo v10, BD Biosciences). A fluorescence minus one (FMO) control was included to delimit the CD16 populations. Gate-selection strategy is described in **Figure S1** in **Supplementary Material 1**.

### 2.8 Statistical Analysis

Statistical analysis was performed with Graph Pad Prism 9 (GraphPad Software, Inc., USA). The normality distribution of variables was evaluated by the Shapiro–Wilk test. Student’s *t*-test was used to compare the normally distributed continuous variables while Mann–Whitney *U*-test and Kruskal-wallis (followed by the *post hoc* Dunn multiple comparisons test) were used to compare non-normally distributed variables. P-values < 0.05 were considered statistically significant.

## 3 Results

### 3.1 Identification and Pathway Enrichment Analysis of Differentially Expressed Genes

**Figure 1:**
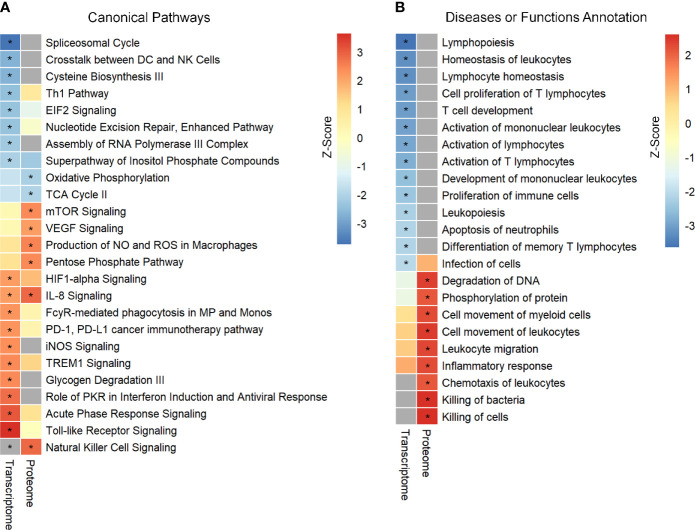
Identification and comparison of pathways enrichment analysis in transcriptome and proteome from septic patients. **(A)** The canonical pathways significantly enriched by DEGs and DEPs. **(B)** Ingenuity biological function activity analysis. * Represents B–H *p*-value <0.05 and a *z*-score >2 (pathway increased) or *z*-score <−2 (pathway decreased). B–H *p*-value were determined using Fischer’s exact test and adjusted for multiple comparisons by Benjamini-Hochberg (BH).

In the present study, two publicly available microarray datasets (GSE65682; Sepsis Dataset 1–SD1 and E‐MTAB‐5273; Sepsis Dataset 2 - SD2) were analyzed, and a total of 2699 DEGs, including 1151 up-regulated and 1548 down-regulated, were selected on the basis of the Venn diagram analysis (**Figure S2** in **Supplementary Material 1** and **Supplementary Material 2**). IPA enrichment analysis for these DEGs identified 18 canonical pathways (CPs) that were significantly enriched. As shown in **Figure 1A** and **Supplementary Material 3**, the application of the IPA *z*‐score algorithm predicted the decrease of eight pathways and increase of 10 pathways. Additionally, IPA analysis predicted 14 diseases or functional annotations that were found to be decreased. Most of these were involved in the proliferation, activation, and homeostasis of lymphocytes (**Figure 1B** and **Supplementary Material 3**).

### 3.2 Differentially Expressed Proteins and Pathway Enrichment Analysis

Proteomics analysis were performed in peripheral blood mononuclear cells (PBMC) samples obtained from 24 septic patients, and resulted in the identification of 2839 proteins, which are presented in a volcano plot (**Figure S3** in **Supplementary Material 1**). This number is in the range of the number of proteins estimated for human PBMC which range from 1738 to 4274 (34–36). We identified 703 proteins as DEPs in the septic patients compared to healthy volunteers, which included 372 up-regulated and 331 down‐regulated proteins. Characteristics of these identified proteins and DEPs are summarized in **Supplementary Material 4**.

Further, enrichment analysis for these 703 DEPs identified eight enriched CPs. Among these, three CPs were characterized by *z*‐score < −2, while six CPs showed *z*‐score > 2 (**Figure 1A** and **Supplementary Material 3**). Additional analysis identified enrichment of nine diseases or functional annotations, most of which were associated with inflammatory response, bacterial killing, and cellular movement (**Figure 1B** and **Supplementary Material 3**). Interestingly, all these nine diseases or functional annotations were found to be increased.

### 3.3 Comparison and Correlations Between Transcriptome and Proteome Data

**Figure 2:**
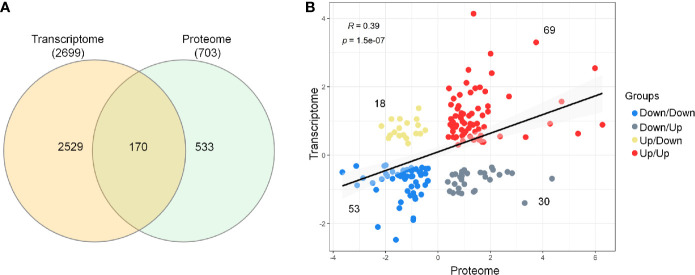
Comparison and correlational analysis between transcriptome and proteome. **(A)** The Venn diagram represents unique and shared genes/proteins between transcriptome and proteome. **(B)** Scatter plot showing the Spearman’s correlation between the mRNA and protein expression levels.

Next, the evaluation of the intersection of the Venn diagram for differentially expressed DEGs and DEPs identified a total of 170 genes/proteins that were co‐differentially expressed in the transcriptome and proteome profiles (**Figure 2A** and **Supplementary Material 5**). These co‐differentially genes/proteins were further divided into four different groups based on the pattern of expression (**Figure 2B**). Two groups were found to be consistent and showed the same pattern of expression for the transcriptome and proteome profiles. These two groups included 69 up/up‐regulated and 53 down/down‐regulated DEGs–DEPs. In comparison to these, the other two groups were characterized by an opposite trend of regulation and included 30 down/up‐regulated and 18 up/down‐regulated DEGs–DEPs. Further, Spearman correlation analysis revealed a low positive correlation value (*R* = 0.39, *p*‐value = 1.5e-07) for the expression of these 170 genes/proteins in the transcriptome and proteome profiles (**Figure 2C**).

### 3.4 Co‐Differentially Expressed Sepsis PPIN

Further, these 170 co-differentially expressed genes/proteins were employed in the construction of sepsis PPIN using the STRING database **(** **Figure S4** in **Supplementary Material 1** **)**. The generated sepsis PPIN consisted of 100 nodes and 271 edges. Further, Markov clustering (MCL) analyses identified seven densely connected regions (modules) that were related to different biological functions. In particular, one module was related to neutrophil degranulation and antimicrobial response, with most genes/proteins up‐regulated. This module included genes/proteins associated with neutrophil collagenase (MMP8), neutrophil subset marker (OLFM4), neutrophil elastase (ELANE), molecules expressed in unstimulated circulating neutrophils (GCA), azurophilic granule proteins (PRTN3 and AZU1), and neutrophil granule proteins (LTF, BPI, and CAMP).

**Figure 3:**
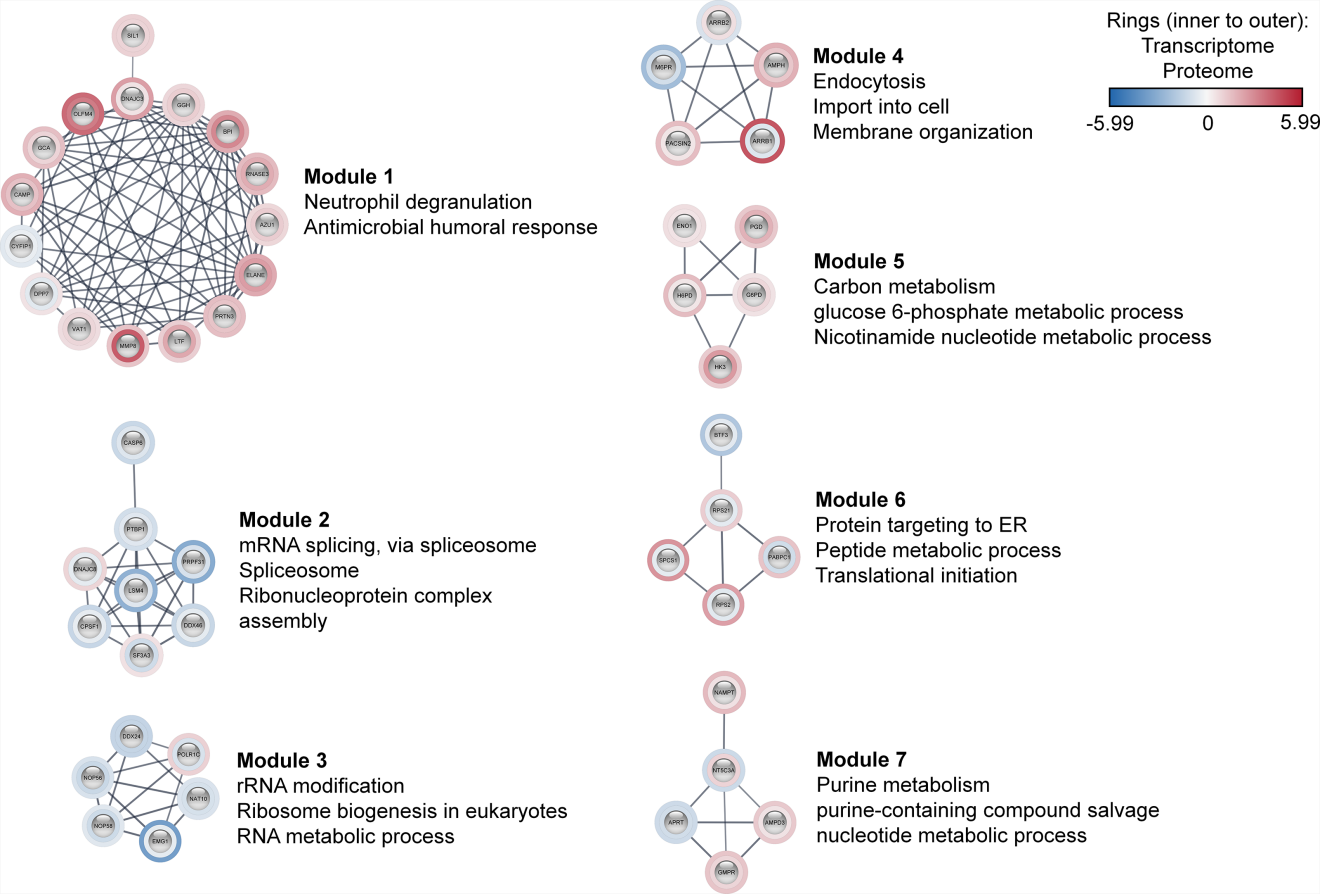
Co-differentially expressed modules in clinical sepsis. Blue nodes represent the down-regulated nodes, and the red nodes represent the up-regulated nodes. Inner rings represent transcriptome data, outer rings represent the proteome data. The modules and their top enriched pathways (FDR-corrected *p*-value <0.05) generated through the STRING enrichment are shown separately.

The present assessment identified three modules that were related to the transfer of information from DNA to mRNA and protein synthesis. In particular, Module 2 mainly included genes/proteins related to mRNA splicing, spliceosome, and ribonucleoprotein complex assembly, whereas Module 3 was associated with rRNA modification, ribosome biogenesis, and RNA metabolic process. These two modules largely included down‐regulated genes/proteins. Module 6 included genes/proteins linked to protein targeting to ER, peptide metabolic processes, and translational initiation. These modules included genes/proteins that are known to play a pivotal role in pre‐mRNA 3′‐end formation (CPSF1), splicing (PRPF31, LSM4, SF3A3, DDX46, and PTBP1), enhancing mRNA stability and translation (NAT10), and ribosomal subunit biogenesis (NOP58, NOP56, and EMG1). Additionally, this set also included an RNA helicase (DDX24) and transcription factor (BTF3). Importantly, all these genes/proteins followed the same expression pattern in both transcriptomic and proteomic profiles. In contrast, signal peptidase complex Subunit 1 (SPCS1), polyadenylate-binding protein 1 (PABPC1), and ribosomal proteins subunits (RPS21 and RPS2) were found to be down‐regulated at transcriptomics level, whereas proteomics profile displayed an up‐regulated expression of these molecules. Additionally, three modules were also found to be related to metabolism (**Figure 3**).

### 3.5 Global Sepsis PPIN and Multiscale Community Detection

**Figure 4:**
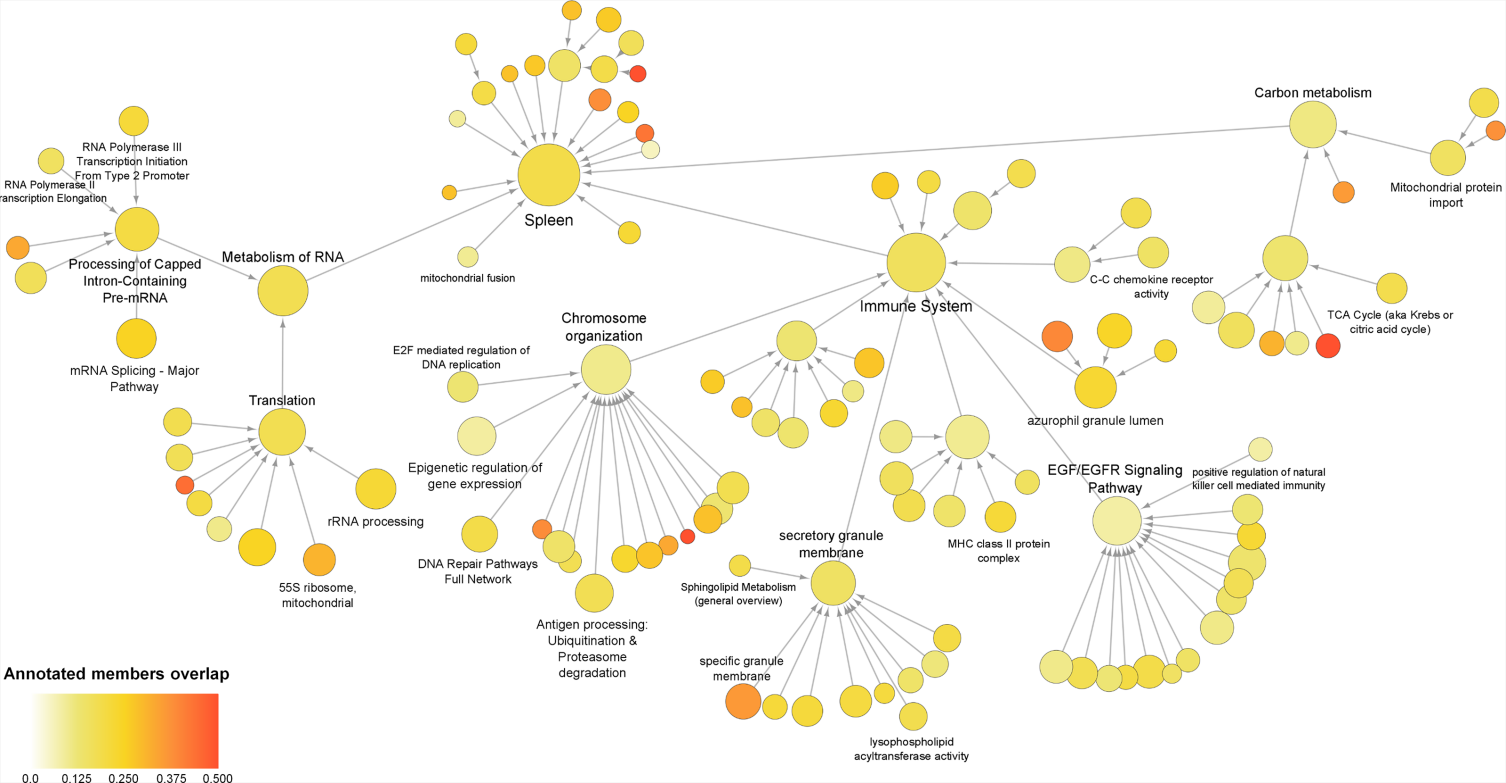
Multiscale community of the global sepsis PPIN. Node size represents the number of genes/proteins annotated in the process. Color scale represents the ratio of overlapping genes in each annotation. Information on these identified communities and annotated members is presented in **Supplemental Material 5**.

To evaluate the occurrence of any other genes/proteins related to the processes that were identified for the co‐differentially expressed modules, a global network was created using all DEGs and DEPs. The global sepsis PPIN comprised 2556 nodes and 26778 edges. Multiscale community analyses for the resulting network revealed 115 enriched communities (**Supplementary Material 5**). For further analysis, certain pathways and processes were selected that were associated with the ones identified in co‐differentially expressed modules **(** **Figure 4** **)**.

Further assessment of the components of the community revealed up‐regulation of genes/proteins associated with the immune system, antigen processing, and azurophil granule lumen. This set included genes/proteins associated with bacterial clearance (CLEC4D), granulopoiesis (MPO), and neutrophil apoptosis (MNDA). Additionally, the set also included neutrophil‐specific matrix metalloproteinase, namely MMP25 and MMP9 (37). Besides this, the analyses also reported down-regulation of genes/proteins related to “C–C chemokine receptor activity”. This group mainly included receptors that regulate neutrophil chemotaxis, namely CCR2, CCR3, CCR4, CCR7, CX3CR1, CXCR3, and CXCR4.

Interestingly, the present analyses reported down‐regulation of the components of the community that were annotated for functions related to transcription and translation, including the metabolism of RNA, mRNA splicing, rRNA biogenesis, epigenetic regulation of gene expression, and 55S mitochondrial ribosome. These communities included 15 eukaryotic initiation factors (eIFs). Among these, 13 eIFs were found to be down‐regulated. These communities also included 25 components of ribosomal subunits (22 down‐regulated) and 24 mitochondrial ribosomal proteins (19 down‐regulated). As well as include exosome complex components proteins, WD-repeat proteins, nucleolar proteins, small nuclear ribonucleoproteins, serine/arginine‐rich splicing factors, pre‐mRNA processing factors, and RNA polymerase subunits. Additionally, the study revealed alteration of carbon metabolism.

### 3.6 Validation of Gene Expression Levels

**Figure 5:**
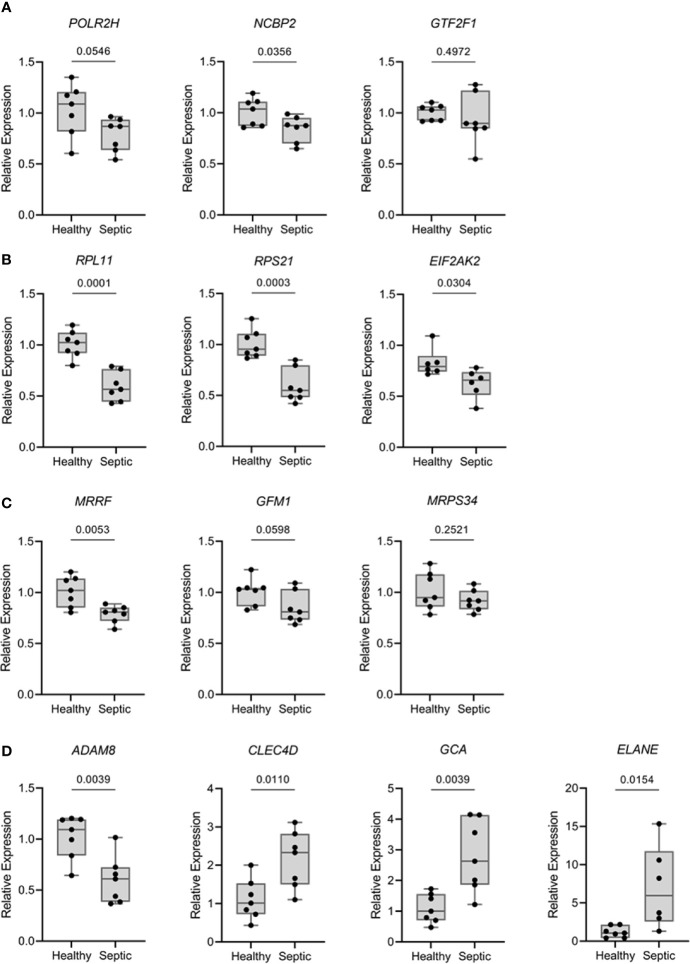
Expression levels of relevant mRNA from over-represented pathways were analyzed by qPCR comparing septic patients (*n* = 7) with healthy volunteers (*n* = 7). Results are expressed as the relative gene expression (2^-∆∆Ct^). **(A)** Genes related with transcriptional processes (*POLR2H*, *NCBP2* and *GTF2F1*). **(B)** Genes related with translational processes (*RPL11*, *RPS21* and *EIF2AK2*). **(C)** Genes related with mitochondrial translation (*MRRF*, *GFM1* and *MRPS34*). **(D)** Genes related with extravasation of leukocytes, killing of bacteria and neutrophils (*ADAM8*, *CLEC4D*, *GCA* and *ELANE*). Data are presented in box plots showing all individual values. *P*-values were determined using student’s t test.

To confirm and validate the aforementioned changes in gene expression, we performed quantitative real-time PCR (qPCR) for PBMCs from patients with sepsis. Thirteen genes were selected based on the results obtained from MCL analyses and/or multiscale community detection and their roles in transcription (mRNA metabolism), translation (mitochondrial translation), and immune response. As illustrated in **Figure 5**, the transcription levels of *RPL11* (ribosomal protein L11), *RPS21* (ribosomal protein S21), *NCBP2* (nuclear cap binding protein subunit 2), and *MRRF* (mitochondrial ribosome recycling factor) were found to be significantly reduced in the sepsis group as compared to the control group (*p*‐value < 0.05). In contrast, a significant increase in the transcription of *CLEC4D* (C-type lectin domain family 4 member D*)*, *GCA* (grancalcin), and *ELANE* (elastase, neutrophil expressed) was reported in the sepsis group as compared to the control. These results were consistent with the findings of the transcriptomic data analysis. Nevertheless, two genes, *EIF2AK2* (eukaryotic translation initiation factor 2 alpha kinase 2) and *ADAM8* (ADAM metallopeptidase domain 8) did not follow the same expression trend. No significant differences were recorded for the expression of *GTF2F1* (general transcription factor IIF subunit 1), *POLR2H (*RNA polymerase II, I and III subunit H), *GFM1* (G elongation factor mitochondrial 1), and *MRPS34* (mitochondrial ribosomal protein S34) between patients and control groups.

### 3.7 Low-Density Neutrophils

The results for proteomics analysis observed in module 1 (**Figure 3**) were obtained from PMBCs isolated from peripheral whole blood. Generally, neutrophils do not fractionate along with PBMCs owing to their higher density as compared to mononuclear cells. However, some low-density neutrophils (LDNs) also known as myeloid‐derived suppressor cells (MDSCs) have been previously shown to get fractionated along with PBMC preparations in certain pathologies, such as coronavirus disease 2019 (COVID‐19) (38, 39), tuberculosis (40) human Ebola virus disease (41), and also sepsis (42, 43). To further support that the source of these genes/proteins found in module 1 were derived from LDNs, we performed flow cytometry with PBMC samples obtained from the same cohort of septic patients included in the proteomic analysis.

**Figure 6:**
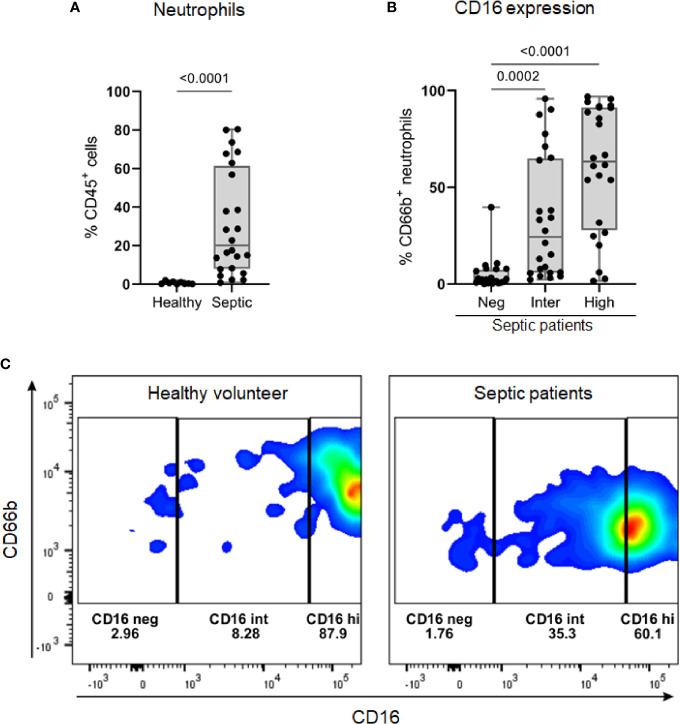
The identification of low-density neutrophil populations in septic patients. **(A)** Percentage of CD66b+ neutrophils, gated on viable CD45+ cells, in PBMC samples from healthy volunteers (*n* = 9) and septic patients (*n* = 24). **(B)** Overall percentage of CD16 populations (negative, intermediate, and high) gated on CD66b+ neutrophils in PBMC from septic patients (*n* = 24). **(C)** Representative dot plots of CD16 populations in CD66b+ neutrophils from one healthy volunteer (left) and one septic patient (right). Data are presented in box plots showing all individual values. *P*-values were determined using Mann–Whitney test **(A)** or Kruskal-Wallis test followed by Dunn’s test **(B)**.

Septic patients presented a higher percentage of neutrophils in Ficoll-isolated PBMC samples than healthy volunteers (**Figure 6A**). On average, a quarter of these neutrophils (median 24.4, IQR: 5.84 – 64.98) showed an intermediate expression level of CD16 (CD16^int^ phenotype) (**Figure 6B**). Among the healthy volunteers, only three had more than 1% of neutrophils in PBMC samples; the percentages of CD16^int^ neutrophils in CD66b+ cells in these subjects were 6.8, 8.28 and 9.75 (representative result in **Figure 6C**).

## 4 Discussion

In general, infections leading to sepsis are caused by various pathogens including bacteria, fungus, viruses, and others. These infections often result in alterations in gene expression, protein-coding, and metabolism (6). In certain cases, these alterations are not finely regulated, and thus lead to organ dysfunction and even death. The present study aimed to analyze these disturbances at the level of gene and protein expression, using pathway enrichment analyses and PPIN.

IPA analysis for the selected DEGs and DEPs resulted in the identification of certain enriched canonical pathways in PBMCs of patients with sepsis that were associated with multiple cellular processes. In particular, these pathways were related to known host responses to sepsis. The results for the gene expression analysis reported in the present study were found to agree with the findings of previous transcriptomic studies. These studies reported an immunosuppressive state in septic patients linked with T lymphocyte-related processes (8, 10, 12). Interestingly, pathways involved in iNOS signaling were found to be increased in patients with sepsis. Previous studies have reported alterations in iNOS expression and activity in response to lipopolysaccharides (LPS) and infection (44). We also found the Toll‐like receptor signaling pathway with an IPA increased status. Generally, Toll‐like receptors are pivotal for host defenses, and tight regulation of these receptors is particularly crucial to prevent hyper inflammation (3).

The results for proteomics analysis further reinforced the idea of modulation of host‐defense pathways in response to sepsis. Signaling pathways involved in the production of nitric oxide (NO) and reactive oxygen species (ROS) in macrophages and Natural Killer (NK) cells were found to be increased. These results are in concordance with previous studies published from our laboratory, which reported that white blood cells of septic patients were characterized by increased NO and ROS production and preserved phagocytic activity (6, 45). In general, NK cells are large granular lymphocytes that are known to be important for host immune response (46, 47). This pattern of defense processes that were predicted to be strongly increased were also observed in the IPA biological function activity (cell movement of leukocytes, leukocyte migration, inflammatory response, chemotaxis of leukocytes, and bacterial killing).

Comparison and correlation of the proteome and transcriptome profiles were performed to investigate which genes/proteins are co-differentially expressed in sepsis. The study revealed co‐differential expression of 170 genes/proteins, with 122 genes/proteins displaying the same expression trends in both transcriptome and proteome profiles. The low to moderate correlation could be attributed to a variety of factors, including translation efficiency, alternative splicing, mRNA stability, folding, assembly, transport and localization, secretion, and degradation (48–50). Since the present study utilized publicly available transcriptomics databases and proteomics results from the current assessment, this 122 co-differentially expressed genes/proteins might be considered as representative of altered biological processes. In previous studies using transcriptomics and proteomics data, obtained from the same sample for both techniques, reported co‐differential expression of genes/proteins in the range of 31–149 (51–53).

The results for MCL analyses emphasized that these co‐differentially expressed genes/proteins played a central role in clinical sepsis. Among the seven modules identified using MCL analyses, Module 1 was found to be associated with neutrophil degranulation and antimicrobial humoral response. In the multiscale community analyses we also highlight the up-regulation of genes/proteins that were related to “Azurophil granule lumen” and “Immune System”. These findings were in concordance with the results for qPCR analysis, which revealed a significant increase in the transcription levels of *CLEC4D*, *GCA*, and *ELANE* in patients with sepsis.

Some of the reported genes/proteins found related to module 1 were previously found in other studies and were described to come from the LDNs that are fractionated along with PBMC (41). To further support that the source of these genes/proteins in our proteomics results comes from LDNs, we performed flow cytometry with PBMC samples obtained from the same cohort of septic patients included in the proteomic analysis. Interestingly, we found a large presence of neutrophils in septic patients when compared to healthy volunteers. As reported, these neutrophils are characterized by the presence of CD16^Int^ to CD16^hi^ expression (42).

Previous studies have suggested that LDNs are associated with the suppression of T cell function and proliferation, similar to the findings of the IPA analysis reported in the present study. Thus, the increase in the number of LDNs might be correlated to the severity of infectious diseases, such as sepsis and COVID‐19 (38, 39, 42, 43). Additionally, these cells are also known to be endowed with a high propensity to spontaneously produce neutrophil extracellular traps (NETs) (41, 54). Particularly, NETs are webs of extracellular DNA decorated with histones, myeloperoxidase, and elastase, which are vital for pathogen clearance. However, excess NET production might induce collateral damage to host tissues during sepsis (55).

Altogether, the results for the present study support the previously established idea that LDNs‐related genes/proteins play a pivotal role in clinical sepsis (43, 56). The study also provided additional evidence for the conservation at the mRNA and protein levels.

According to the central dogma of molecular biology, genes that encode proteins are first transcribed into mRNAs, which are further translated into proteins (57). Several molecular events are involved in the regulation of this transfer of information (58). In particular, genes/proteins present in Modules 2, 3, and 6 are related to the processes of transcription and translation. Interestingly, the results for IPA analysis predicted down-regulation of the eukaryotic initiation factor 2 (eIF2) signaling pathway in both proteomics (*z*‐score = −1.00 and −log10 FDR = 4.48) and transcriptomics analysis (*z*‐score = −2.40 and −log10 FDR = 2.91). This pathway plays a crucial key role in global and specific mRNA translation processes. In particular, the phosphorylation of eIF2 is known to inhibit eIF2B, which is further related to translational initiation (59, 60).

Assessment using multiscale community analyses further resulted in the identification of 13 eIFs down‐regulated and 22 components of ribosomal subunits with down‐regulated expression in the community related to translation. The present study also revealed that communities related to “rRNA processing” and “mRNA Splicing” are largely represented by down‐regulated genes/proteins. The results for qPCR analysis partially validated the findings of the transcriptomic analysis. In particular, the qPCR analysis showed significant down-regulation of *RPL11*, *RPS21*, *NCBP2*, and *MRRF* during sepsis. Nonetheless, results for *EIF2AK2* expression were found to be contradictory in these two analyses.

Altogether, these results are suggestive of impairment of the translation process in peripheral cells of patients with sepsis. These results are supported by the translatome analysis performed for the kidney obtained from an animal model of endotoxemia, wherein the EIF2AK2/eIF2α axis was identified as the key mediator of translation initiation block in late‐phase sepsis (61). These results were also found to be in concordance with the findings of Calvano, Xiao (62). The study showed that the response of human blood leukocytes to acute systemic inflammation, induced by LPS, involved concerted dysregulation of functional modules in mitochondrial bioenergetics, protein synthesis, and protein degradation. Thus, these results reinforced the idea of reprioritization of the leukocytic transcription regulatory program in response to endotoxin (62).

In addition to this, multiscale community analyses revealed that the community related to translation included 19 down‐regulated mitochondrial ribosomal proteins. This community also involved genes/proteins related to the 55S mitochondrial ribosome. In particular, these mitoribosomes are known to be responsible for the translation of essential components of the complexes involved in oxidative phosphorylation (63). Therefore, down‐regulation of mitoribosomes might be directly involved in the remarkable mitochondrial dysfunction, observed during sepsis (64).

The present study was associated with certain limitations. For analysis, the transcriptome data were obtained from two publicly available microarray datasets for septic patients, whereas the proteome data were obtained from the cohort of patients assessed in the present study. Thus, the transcriptome and proteome data used in the present analyses were obtained from different sets of patients. Although, the number of co‐differentially expressed genes/proteins identified in the present study were higher as compared to those reported in previous studies (51–53). We select the DEPs based on P and Log2FC values (27, 28). Adjustment for the Benjamini-Hochberg methods would lead to lower number of DEPs, but without changing the conclusions. Overall, the pathways related to the ROS, NO and phagocytosis were consistent with the findings of previous studies conducted by our group and other researchers, which involved peripheral blood cells obtained from septic patients.

**Figure 7:**
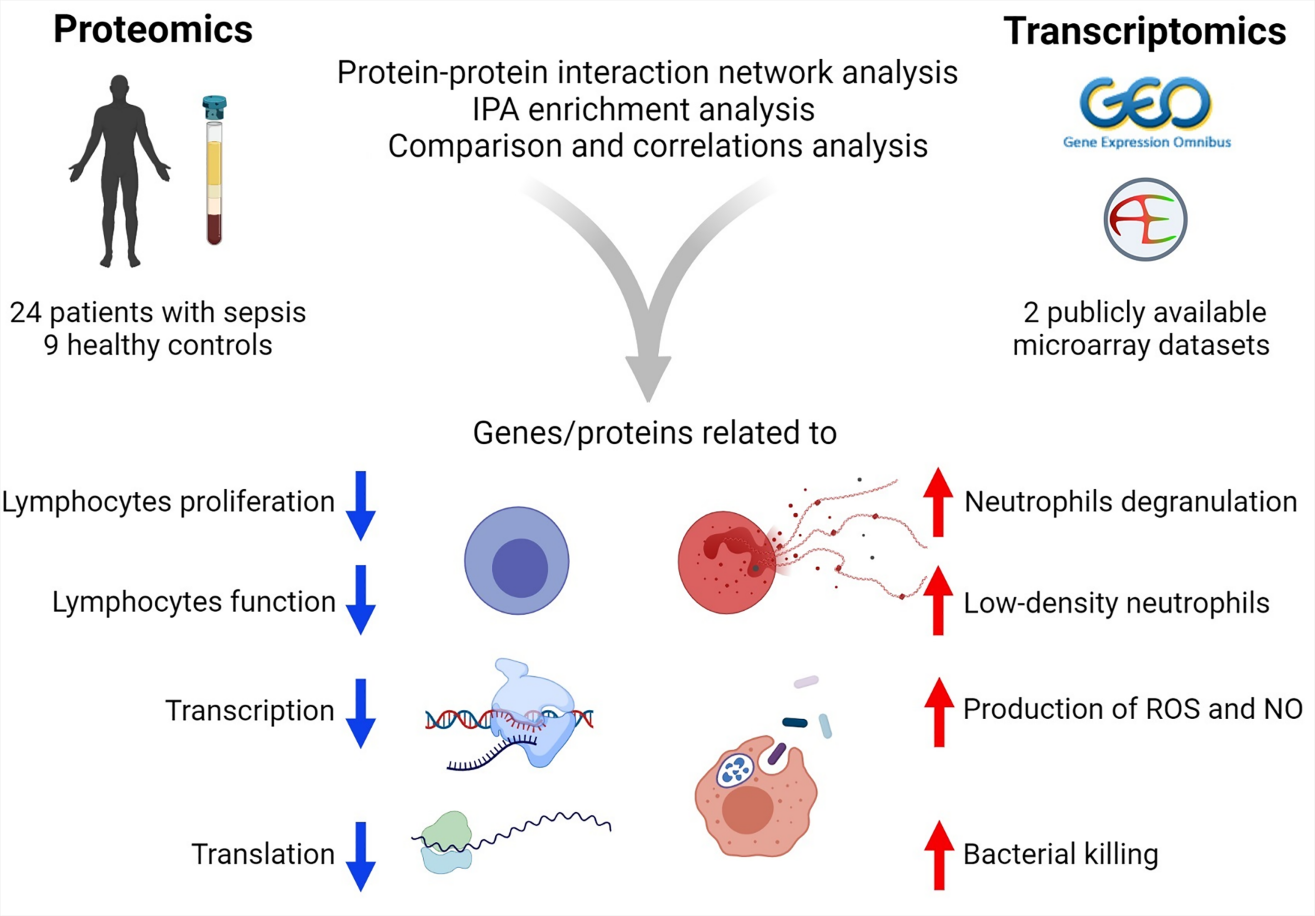
Summary of the results of transcriptomics and proteomics profiling of septic patients.

In summary (**Figure 7**), the present study utilized a combination of transcriptomic and proteomic data and systems biology approaches to provide better insights into the host defense mechanisms against sepsis. In particular, the study identified 170 co‐differentially expressed genes/proteins. Among these, a set of 122 genes/proteins displayed similar expression trends in the transcriptome and proteome profiles. Interestingly, these genes/proteins were found to be associated with hosts’ responses during the early stages of sepsis. Further, PPIN analyses revealed the involvement of modules related to the reprioritization of biological functions in response to sepsis, *via* a transcriptional and translational shutdown, except for an up-regulated set of genes/proteins that were related to low-density neutrophils and host‐defense system.

## Data Availability Statement

The microarray datasets for this manuscript are freely available with accession numbers GSE65682 (Gene Expression Omnibus) and E-MTAB-5273 (EBI ArrayExpress). Mass spectrometry data were deposited to the ProteomeXchange Consortium *via* the MassIVE partner repository with the dataset identifier MSV000087733.

## Ethics Statement

The studies involving human participants were reviewed and approved by University Ethical Committee (CEP/UNIFESP 1171/2017). The patients/participants provided their written informed consent to participate in this study.

## Author Contributions

GL, Data analysis, curation and visualization, methodology, writing and review. BF, Proteomic samples preparation, qPCR experiments, flow cytometry analysis, writing and review. RS, Supervision, conceptualization, writing and review. AT and EN, performed the mass spectrometry-based data acquisition, processed the mass spectrometry data and review. MC, Flow cytometry data acquisition, analysis and review. TP and BS, Methodology and review. EC-N, Bioinformatics platform support for analysis and review. MA, LA, and FF, contributed to the design of the study, the selection, enrollment, and monitoring of patients. All authors contributed to the article and approved the submitted version.

## Funding

This work was funded by FAPESP (2017/21052-0) to RS. GL has a scholarship from FAPESP (2019/20532-3). BF has a scholarship from FAPESP (2016/13855-2).

## Conflict of Interest

The authors declare that the research was conducted in the absence of any commercial or financial relationships that could be construed as a potential conflict of interest.

## Publisher’s Note

All claims expressed in this article are solely those of the authors and do not necessarily represent those of their affiliated organizations, or those of the publisher, the editors and the reviewers. Any product that may be evaluated in this article, or claim that may be made by its manufacturer, is not guaranteed or endorsed by the publisher.

## Supplementary Material

Supplementary Material 1 Supplementary Methods, figures, and tables.

Supplementary Material 2 Differentially expressed genes.

Supplementary Material 3 Differentially expressed protein.

Supplementary Material 4 Ingenuity pathway analysis.

Supplementary Material 5 Merge of DEGs and DEPs, and Community Information.

## References

[1] Singer M Deutschman CS Seymour CW Shankar-Hari M Annane D Bauer M. The Third International Consensus Definitions for Sepsis and Septic Shock (Sepsis-3). JAMA (2016) 315(8):801–10. doi: 10.1001/jama.2016.0287 PMC4968574 26903338

[2] Berg D Gerlach H. Recent Advances in Understanding and Managing Sepsis. F1000Res (2018) 7:F1000 Faculty Rev–570. doi: 10.12688/f1000research.15758.1

[3] Salomao R Brunialti MK Rapozo MM Baggio-Zappia GL Galanos C Freudenberg M. Bacterial Sensing, Cell Signaling, and Modulation of the Immune Response During Sepsis. Shock (2012) 38(3):227–42. doi: 10.1097/SHK.0b013e318262c4b0 22777111

[4] van der Poll T van de Veerdonk FL Scicluna BP Netea MG. The Immunopathology of Sepsis and Potential Therapeutic Targets. Nat Rev Immunol (2017) 17(7):407–20. doi: 10.1038/nri.2017.36 28436424

[5] Hotchkiss RS Monneret G Payen D. Immunosuppression in Sepsis: A Novel Understanding of the Disorder and a New Therapeutic Approach. Lancet Infect Dis (2013) 13(3):260–8. doi: 10.1016/S1473-3099(13)70001-X PMC3798159 23427891

[6] Salomão R Ferreira BL Salomão MC Santos SS Azevedo LCP Brunialti MKC. Sepsis: Evolving Concepts and Challenges. Braz J Med Biol Res (2019) 52(4):e8595. doi: 10.1590/1414-431X20198595 30994733 PMC6472937

[7] Sharma NK Tashima AK Brunialti MKC Ferreira ER Torquato RJS Mortara RA. Proteomic Study Revealed Cellular Assembly and Lipid Metabolism Dysregulation in Sepsis Secondary to Community-Acquired Pneumonia. Sci Rep (2017) 7(1):15606. doi: 10.1038/s41598-017-15755-1 29142235 PMC5688086

[8] Xiao W Mindrinos MN Seok J Cuschieri J Cuenca AG Gao H. A Genomic Storm in Critically Injured Humans. J Exp Med (2011) 208(13):2581–90. doi: 10.1084/jem.20111354 PMC3244029 22110166

[9] Scicluna BP van Vught LA Zwinderman AH Wiewel MA Davenport EE Burnham KL. Classification of Patients With Sepsis According to Blood Genomic Endotype: A Prospective Cohort Study. Lancet Respir Med (2017) 5(10):816–26. doi: 10.1016/S2213-2600(17)30294-1 28864056

[10] Davenport EE Burnham KL Radhakrishnan J Humburg P Hutton P Mills TC. Genomic Landscape of the Individual Host Response and Outcomes in Sepsis: A Prospective Cohort Study. Lancet Respir Med (2016) 4(4):259–71. doi: 10.1016/S2213-2600(16)00046-1 PMC4820667 26917434

[11] Burnham KL Davenport EE Radhakrishnan J Humburg P Gordon AC Hutton P. Shared and Distinct Aspects of the Sepsis Transcriptomic Response to Fecal Peritonitis and Pneumonia. Am J Respir Crit Care Med (2017) 196(3):328–39. doi: 10.1164/rccm.201608-1685OC PMC5549866 28036233

[12] Almansa R Heredia-Rodríguez M Gomez-Sanchez E Andaluz-Ojeda D Iglesias V Rico L. Transcriptomic Correlates of Organ Failure Extent in Sepsis. J Infect (2015) 70(5):445–56. doi: 10.1016/j.jinf.2014.12.010 25557485

[13] Leite GGF Scicluna BP van der Poll T Salomão R. Genetic Signature Related to Heme-Hemoglobin Metabolism Pathway in Sepsis Secondary to Pneumonia. NPJ Syst Biol Appl (2019) 5(1):26. doi: 10.1038/s41540-019-0105-4 31396396 PMC6672010

[14] Sharma NK Ferreira BL Tashima AK Brunialti MKC Torquato RJS Bafi A. Lipid Metabolism Impairment in Patients With Sepsis Secondary to Hospital Acquired Pneumonia, a Proteomic Analysis. Clin Proteomics (2019) 16:29. doi: 10.1186/s12014-019-9252-2 31341447 PMC6631513

[15] Hu Y Cheng L Zhong W Chen M Zhang Q. Bioinformatics Analysis of Gene Expression Profiles for Risk Prediction in Patients With Septic Shock. Med Sci Monit (2019) 25:9563–71. doi: 10.12659/msm.918491 PMC6929537 31838482

[16] Zhai J Qi A Zhang Y Jiao L Liu Y Shou S. Bioinformatics Analysis for Multiple Gene Expression Profiles in Sepsis. Med Sci Monit (2020) 26:e920818. doi: 10.12659/msm.920818 32280132 PMC7171431

[17] Hu Y Zhong W Chen M Zhang Q. Identifying Crucial Genes for Prognosis in Septic Patients: Gene Integration Study Based on PRISMA Guidelines. Medicine (2019) 98(33):e16807. doi: 10.1097/md.0000000000016807 31415393 PMC6831352

[18] Khan HN Perlee D Schoenmaker L van der Meer A-J Franitza M Toliat MR. Leukocyte Transcriptional Signatures Dependent on LPS Dosage in Human Endotoxemia. J Leukoc Biol (2019) 106(5):1153–60. doi: 10.1002/JLB.4A0219-050R PMC6852106 31280495

[19] Bone RC Balk RA Cerra FB Dellinger RP Fein AM Knaus WA. Definitions for Sepsis and Organ Failure and Guidelines for the Use of Innovative Therapies in Sepsis. The ACCP/SCCM Consensus Conference Committee. American College of Chest Physicians/Society of Critical Care Medicine. Chest (1992) 101(6):1644–55. doi: 10.1378/chest.101.6.1644 1303622

[20] Bradford MM. A Rapid and Sensitive Method for the Quantitation of Microgram Quantities of Protein Utilizing the Principle of Protein-Dye Binding. Anal Biochem (1976) 72:248–54. doi: 10.1006/abio.1976.9999 942051

[21] Distler U Kuharev J Navarro P Levin Y Schild H Tenzer S. Drift Time-Specific Collision Energies Enable Deep-Coverage Data-Independent Acquisition Proteomics. Nat Methods (2014) 11(2):167–70. doi: 10.1038/nmeth.2767 24336358

[22] Pedroso AP Souza AP Dornellas AP Oyama LM Nascimento CM Santos GM. Intrauterine Growth Restriction Programs the Hypothalamus of Adult Male Rats: Integrated Analysis of Proteomic and Metabolomic Data. J Proteome Res (2017) 16(4):1515–25. doi: 10.1021/acs.jproteome.6b00923 28314371

[23] Abreu TF Sumitomo BN Nishiyama MY Jr. Oliveira UC Souza GH Kitano ES. Peptidomics of Acanthoscurria Gomesiana Spider Venom Reveals New Toxins With Potential Antimicrobial Activity. J Proteomics (2017) 151:232–42. doi: 10.1016/j.jprot.2016.07.012 27436114

[24] Câmara GA Nishiyama-Jr MY Kitano ES Oliveira UC Silva PID Junqueira-de-Azevedo IL. A Multiomics Approach Unravels New Toxins With Possible *In Silico* Antimicrobial, Antiviral, and Antitumoral Activities in the Venom of Acanthoscurria Rondoniae. Front Pharmacol (2020) 11:1075. doi: 10.3389/fphar.2020.01075 32774304 PMC7388414

[25] Silva JC Gorenstein MV Li G-Z Vissers JPC Geromanos SJ. Absolute Quantification of Proteins by LCMSE: A Virtue of Parallel MS Acquisition. Mol Cell Proteomics (2006) 5(1):144–56. doi: 10.1074/mcp.M500230-MCP200 16219938

[26] Tyanova S Temu T Sinitcyn P Carlson A Hein MY Geiger T. The Perseus Computational Platform for Comprehensive Analysis of (Prote)Omics Data. Nat Methods (2016) 13(9):731–40. doi: 10.1038/nmeth.3901 27348712

[27] Tiwari NK Sathyanesan M Kumar V Newton SS. A Comparative Analysis of Erythropoietin and Carbamoylated Erythropoietin Proteome Profiles. Life (2021) 11(4):359. doi: 10.3390/life11040359 33921564 PMC8073529

[28] Zhang Q Ma C Gearing M Wang PG Chin L-S Li L. Integrated Proteomics and Network Analysis Identifies Protein Hubs and Network Alterations in Alzheimer’s Disease. Acta Neuropathol Commun (2018) 6(1):19. doi: 10.1186/s40478-018-0524-2 29490708 PMC5831854

[29] Legeay M Doncheva N Morris J Jensen L. Visualize Omics Data on Networks With Omics Visualizer, A Cytoscape App. F1000Res (2020) 9(157)1–17. doi: 10.12688/f1000research.22280.2 PMC7194485 32399202

[30] Doncheva NT Morris JH Gorodkin J Jensen LJ. Cytoscape Stringapp: Network Analysis and Visualization of Proteomics Data. J Proteome Res (2019) 18(2):623–32. doi: 10.1021/acs.jproteome.8b00702 PMC6800166 30450911

[31] Singhal A Cao S Churas C Pratt D Fortunato S Zheng F. Multiscale Community Detection in Cytoscape. PloS Comput Biol (2020) 16(10):e1008239. doi: 10.1371/journal.pcbi.1008239 33095781 PMC7584444

[32] Tanaka A To J O’Brien B Donnelly S Lund M. Selection of Reliable Reference Genes for the Normalisation of Gene Expression Levels Following Time Course LPS Stimulation of Murine Bone Marrow Derived Macrophages. BMC Immunol (2017) 18(1):43–. doi: 10.1186/s12865-017-0223-y PMC5627409 28974200

[33] Gritte RB Souza-Siqueira T Mais LN. Reference Genes for Quantitative Qpcr Analyses in Monocytes of Septic Patients (2020) (Accessed April 15, 2021).

[34] Li J Guo M Tian X Wang X Yang X Wu P. Virus-Host Interactome and Proteomic Survey Reveal Potential Virulence Factors Influencing SARS-Cov-2 Pathogenesis. Med (2021) 2(1):99–112.e7. doi: 10.1016/j.medj.2020.07.002 32838362 PMC7373048

[35] Chaves DFS Carvalho PC Brasili E Rogero MM Hassimotto NA Diedrich JK. Proteomic Analysis of Peripheral Blood Mononuclear Cells After a High-Fat, High-Carbohydrate Meal With Orange Juice. J Proteome Res (2017) 16(11):4086–92. doi: 10.1021/acs.jproteome.7b00476 28927270

[36] Zhang L Wang Z Chen Y Zhang C Xie S Cui Y. Label-Free Proteomic Analysis of Pbmcs Reveals Gender Differences in Response to Long-Term Antiretroviral Therapy of HIV. J Proteomics (2015) 126:46–53. doi: 10.1016/j.jprot.2015.05.033 26045010

[37] Steichen AL Binstock BJ Mishra BB. Sharma J. C-Type Lectin Receptor Clec4d Plays a Protective Role in Resolution of Gram-Negative Pneumonia. J Leukoc Biol (2013) 94(3):393–8. doi: 10.1189/jlb.1212622 PMC3747124 23709686

[38] Agrati C Sacchi A Bordoni V Cimini E Notari S Grassi G. Expansion of Myeloid-Derived Suppressor Cells in Patients With Severe Coronavirus Disease (COVID-19). Cell Death Differ (2020) 27(11):3196–207. doi: 10.1038/s41418-020-0572-6 PMC7278239 32514047

[39] Morrissey SM Geller AE Hu X Tieri D Ding C Klaes CK. A Specific Low-Density Neutrophil Population Correlates With Hypercoagulation and Disease Severity in Hospitalized COVID-19 Patients. JCI Insight (2021) 6(9):e148435. doi: 10.1172/jci.insight.148435 PMC8262329 33986193

[40] Deng Y Ye J Luo Q Huang Z Peng Y Xiong G. Low-Density Granulocytes are Elevated in Mycobacterial Infection and Associated With the Severity of Tuberculosis. PloS One (2016) 11(4):e0153567–e. doi: 10.1371/journal.pone.0153567 PMC4830625 27073889

[41] Eisfeld AJ Halfmann PJ Wendler JP Kyle JE Burnum-Johnson KE Peralta Z. Multi-Platform 'Omics Analysis of Human Ebola Virus Disease Pathogenesis. Cell Host Microbe (2017) 22(6):817–29.e8. doi: 10.1016/j.chom.2017.10.011 29154144 PMC5730472

[42] Darcy CJ Minigo G Piera KA Davis JS McNeil YR Chen Y. Neutrophils With Myeloid Derived Suppressor Function Deplete Arginine and Constrain T Cell Function in Septic Shock Patients. Crit Care (2014) 18(4):R163–R. doi: 10.1186/cc14003 PMC4261583 25084831

[43] Uhel F Azzaoui I Grégoire M Pangault C Dulong J Tadié JM. Early Expansion of Circulating Granulocytic Myeloid-Derived Suppressor Cells Predicts Development of Nosocomial Infections in Patients With Sepsis. Am J Respir Crit Care Med (2017) 196(3):315–27. doi: 10.1164/rccm.201606-1143OC 28146645

[44] Cyr A Chambers L Waltz PK Whelan SP Kohut L Carchman E. Endotoxin Engages Mitochondrial Quality Control *via* an Inos-Reactive Oxygen Species Signaling Pathway in Hepatocytes. Oxid Med Cell Longev (2019) 2019:4745067–. doi: 10.1155/2019/4745067 PMC6854992 31772705

[45] Santos SS Carmo AM Brunialti MKC Machado FR Azevedo LC Assunção M. Modulation of Monocytes in Septic Patients: Preserved Phagocytic Activity, Increased ROS and NO Generation, and Decreased Production of Inflammatory Cytokines. Intensive Care Med Exp (2016) 4:5. doi: 10.1186/s40635-016-0078-1 26879814 PMC4754229

[46] Guo Y Patil NK Luan L Bohannon JK Sherwood ER. The Biology of Natural Killer Cells During Sepsis. Immunology (2018) 153(2):190–202. doi: 10.1111/imm.12854 29064085 PMC5765373

[47] Chiche L Forel J-M Thomas G Farnarier C Vely F Bléry M. The Role of Natural Killer Cells in Sepsis. J BioMed Biotechnol (2011) 2011:986491. doi: 10.1155/2011/986491 21629707 PMC3100670

[48] Wang X Li D Song S Zhang Y Li Y Wang X. Combined Transcriptomics and Proteomics Forecast Analysis for Potential Genes Regulating the Columbian Plumage Color in Chickens. PloS One (2019) 14(11):e0210850–e. doi: 10.1371/journal.pone.0210850 PMC6834273 31693656

[49] Greenbaum D Jansen R Gerstein M. Analysis of Mrna Expression and Protein Abundance Data: An Approach for the Comparison of the Enrichment of Features in the Cellular Population of Proteins and Transcripts. Bioinformatics (2002) 18(4):585–96. doi: 10.1093/bioinformatics/18.4.585 12016056

[50] Xie J Zhao Y Dong N Tian X Feng J Liu P. Proteomics and Transcriptomics Jointly Identify the Key Role of Oxidative Phosphorylation in Fluoride-Induced Myocardial Mitochondrial Dysfunction in Rats. Ecotoxicol Environ Saf (2021) 218:112271. doi: 10.1016/j.ecoenv.2021.112271 33932654

[51] Ding H Mo S Qian Y Yuan G Wu X Ge C. Integrated Proteome and Transcriptome Analyses Revealed Key Factors Involved in Tomato (Solanum Lycopersicum) Under High Temperature Stress. Food Energy Secur (2020) 9(4):e239. doi: 10.1002/fes3.239

[52] Chen B Wang D Bian Y Li J Yang T Li N. Systematic Identification of Hub Genes in Placenta Accreta Spectrum Based on Integrated Transcriptomic and Proteomic Analysis. Front Genet (2020) 11:551495. doi: 10.3389/fgene.2020.551495 33101378 PMC7522549

[53] Liu R Xu B Yu S Zhang J Sun H Liu C. Integrated Transcriptomic and Proteomic Analyses of the Interaction Between Chicken Synovial Fibroblasts and Mycoplasma Synoviae. Front Microbiol (2020) 11:576. doi: 10.3389/fmicb.2020.00576 32318048 PMC7147270

[54] Reusch N De Domenico E Bonaguro L Schulte-Schrepping J Baßler K Schultze JL. Neutrophils in COVID-19. Front Immunol (2021) 12:652470. doi: 10.3389/fimmu.2021.652470 33841435 PMC8027077

[55] Denning N-L Aziz M Gurien SD Wang P. Damps and Nets in Sepsis. Front Immunol (2019) 10:2536. doi: 10.3389/fimmu.2019.02536 31736963 PMC6831555

[56] Alder MN Opoka AM Lahni P Hildeman DA Wong HR. Olfactomedin-4 Is a Candidate Marker for a Pathogenic Neutrophil Subset in Septic Shock. Crit Care Med (2017) 45(4):e426–e32. doi: 10.1097/CCM.0000000000002102 PMC5512699 27635771

[57] Crick F. Central Dogma of Molecular Biology. Nature (1970) 227(5258):561–3. doi: 10.1038/227561a0 4913914

[58] Maier T Güell M Serrano L. Correlation of Mrna and Protein in Complex Biological Samples. FEBS Lett (2009) 583(24):3966–73. doi: 10.1016/j.febslet.2009.10.036 19850042

[59] Proud CG. Eif2 and the Control of Cell Physiology. Semin Cell Dev Biol (2005) 16(1):3–12. doi: 10.1016/j.semcdb.2004.11.004 15659334

[60] Wek RC. Role of Eif2α Kinases in Translational Control and Adaptation to Cellular Stress. Cold Spring Harb Perspect Biol (2018) 10(7):a032870. doi: 10.1101/cshperspect.a032870 29440070 PMC6028073

[61] Hato T Maier B Syed F Myslinski J Zollman A Plotkin Z. Bacterial Sepsis Triggers an Antiviral Response That Causes Translation Shutdown. J Clin Invest (2019) 129(1):296–309. doi: 10.1172/JCI123284 30507610 PMC6307966

[62] Calvano SE Xiao W Richards DR Felciano RM Baker HV Cho RJ. A Network-Based Analysis of Systemic Inflammation in Humans. Nature (2005) 437(7061):1032–7. doi: 10.1038/nature03985 16136080

[63] Kaushal PS Sharma MR Agrawal RK. The 55S Mammalian Mitochondrial Ribosome and Its Trna-Exit Region. Biochimie (2015) 114:119–26. doi: 10.1016/j.biochi.2015.03.013 PMC4772884 25797916

[64] Singer M. The Role of Mitochondrial Dysfunction in Sepsis-Induced Multi-Organ Failure. Virulence (2014) 5(1):66–72. doi: 10.4161/viru.26907 24185508 PMC3916385

