# Supplementary material for: Combined Transcriptome and Proteome Leukocyte’s Profiling Reveals Up-Regulated Module of Genes/Proteins Related to Low Density Neutrophils and Impaired Transcription and Translation Processes in Clinical Sepsis
Source: Front Immunol. 2021 Sep 10;12:744799. doi: 10.3389/fimmu.2021.744799 (PMC8477441; doi:10.3389/fimmu.2021.744799)
Supplement: Supplementary Material 1 — Supplementary Methods, figures, and tables. [file DataSheet_1.docx]

**Supplementary Material 1**

**Supplementary Methods:** additional information about datasets and analyzes

*GSE65682 (renamed to Sepsis Dataset 1; SD1)*

The S1 dataset originally consists of 760 samples from adult patients admitted to Netherlands ICUs with sepsis due to community acquired pneumonia, hospital acquired pneumonia, hospitalized patients without infection, and 42 samples from healthy subjects (controls). Whole blood samples were collected using the PAXgene tubes (Becton-Dickinson, Breda, the Netherlands), RNA was isolated using the PAXgene blood mRNA kit (Qiagen, Venlo, the Netherlands) and then microarray analysis was performed using the Affymetrix Human Genome U219 array platform and deposited in GEO under the accession number [GSE65682](https://www.ncbi.nlm.nih.gov/geo/query/acc.cgi?acc=GSE65682).

For this dataset, we work with the “GSE65682_series_matrix.txt”, related to “The Molecular Diagnosis and Risk Stratification of Sepsis (MARS) consortium”. We used the patients from the “Classification of patients with sepsis according to blood genomic endotype: a prospective cohort study” as reference for selecting the group of septic patients, less sample "GSM1692240" because it is from a < 18 years old individual. This file was previously processed following the steps: Pre-processing and quality control was performed by using the Affy package version 1.36.1. Array data were background corrected by Robust Multi-array Average, quantiles-normalized and summarized by medianpolish using the expresso function. The resultant 49,386 probe intensities were filtered by means of a 0.5 variance cutoff using the genefilter method to recover 24,646 expressed probes. The occurrence of non-experimental chip effects was evaluated by means of the Surrogate Variable Analysis (R package version 3.4.0) and corrected by the empirical Bayes method ComBat.

Therefore, we used 478 septic patients and 42 healthy controls to detect differentially expressed genes between sepsis and controls.

*E-MTAB-5273 (SD2)*

The S2 dataset originally consists of 227 samples from adult patients admitted to United Kingdom ICUs with sepsis due to community acquired pneumonia or faecal peritonitis and 10 samples of patients scheduled for heart surgery (controls). Leukocyte samples were obtained using the LeukoLOCK filters (Life Technologies, Carlsbad, CA) and subsequently the microarray analysis was performed using the Illumina HumanHT-12 v4 Expression BeadChips platform and deposited on the ArrayExpress under accession number E-MTAB-5273.

For this dataset, we work with the “Burnham_sepsis_discovery_normalised_231.txt”, related to publication “Shared and distinct aspects of the sepsis transcriptomic response to fecal peritonitis and pneumonia”. This file contains 231 samples, 46 from sepsis patients collected within the first 24 hours, 175 from sepsis patients collected after 24 hours and 10 controls.

This file was previously processed following the steps: Background signals were subtracted. Six samples were identified as outliers and removed (FP0035.3, FP0046.5, CAP0056.B.5, CAP0140.B.5, CAP0383.5, FP0058.B.1). The raw data were restricted to probes that had a detection value greater than or equal to 0.95 in at least 5% of samples (27,159 probes) and normalised using the Variance Stabilisation and Normalisation method.” (<https://www.ebi.ac.uk/arrayexpress/experiments/E-MTAB-5273/protocols/>)

Therefore, we get 46 septic patients and 10 healthy controls to detect differentially expressed genes between sepsis and controls.

**
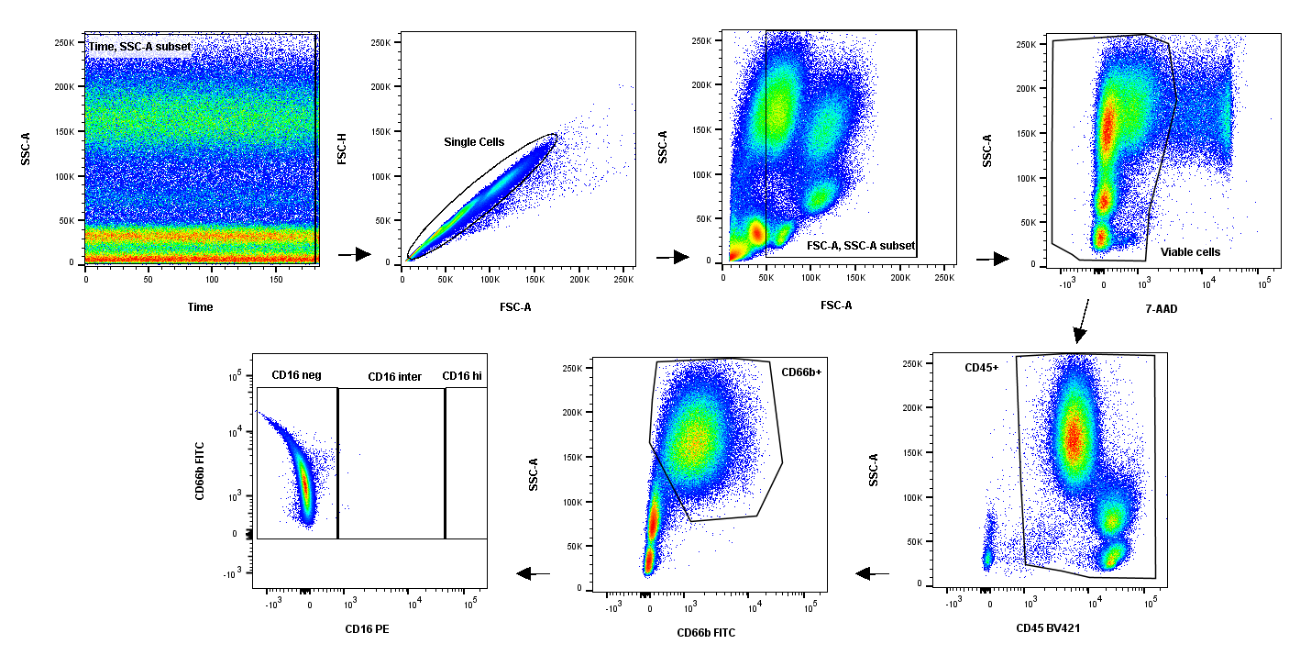
Supplementary Figures**

**Figure S1. Gate-selection strategy for the immunophenotyping of low-density neutrophils.** CD66b^+^ neutrophil population was defined from live CD45^+^ cells after a selection of single cells and removal of debris in the forward versus side scatter gating. CD16 expression was then evaluated within CD66b^+^ neutrophils using a fluorescence minus one (FMO) control.


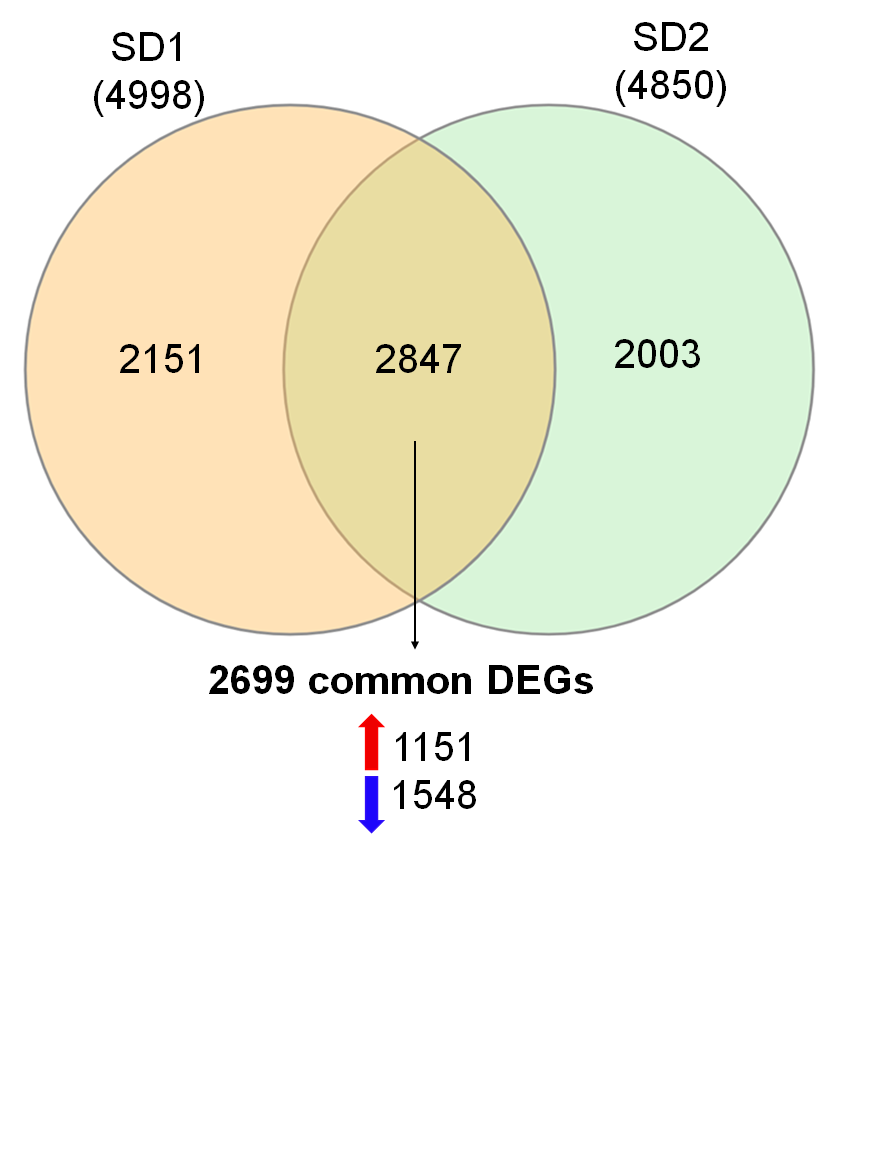


**Figure S2** - Venn diagram demonstrating the overlap between the genes in the two microarray datasets.


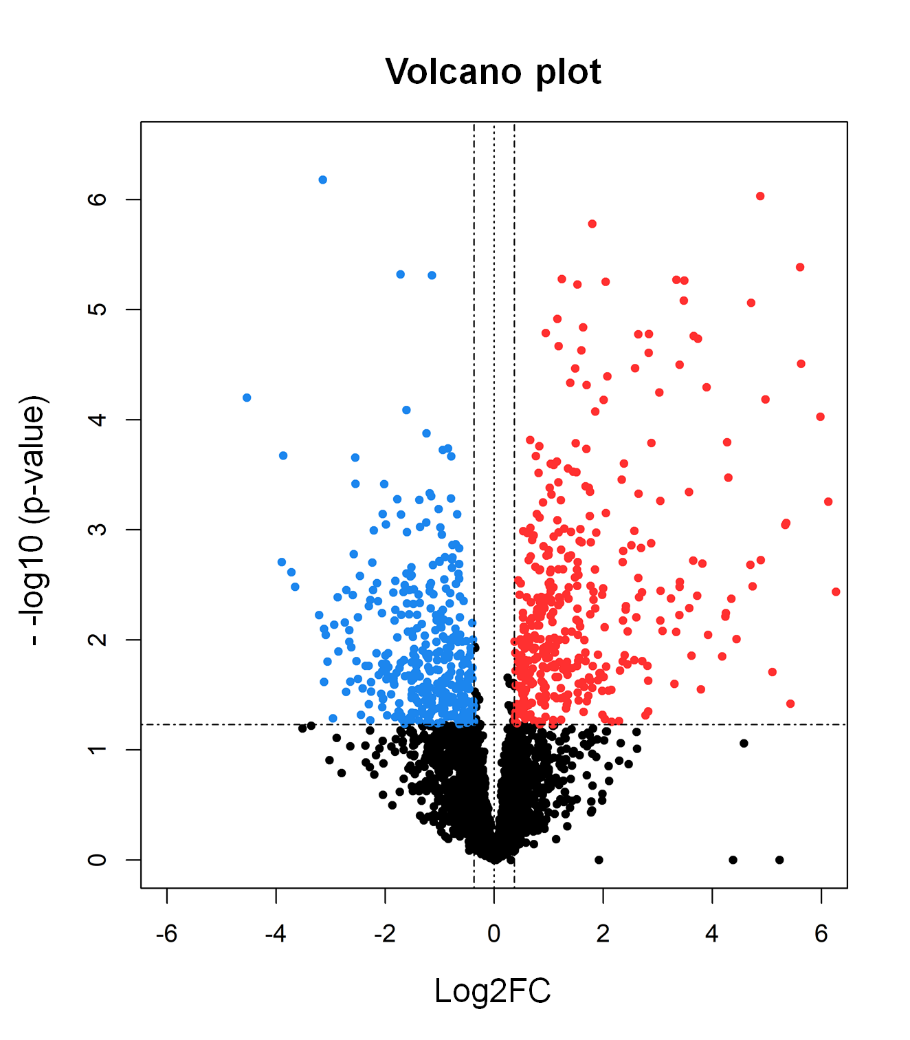


**Figure S3** - Volcano plot of DEPs in sepsis. Dots represent different proteins, and the black dots mean proteins without significant different expression. Red dots (Up) represent significantly up-regulated proteins (P < 0.05 and Log_2_FC > 0.37) in sepsis, and the blue dots mean significantly down-r​e​g​u​l​a​t​e​d​ proteins (P < 0.05 and Log_2_FC < -0.37) in sepsis.


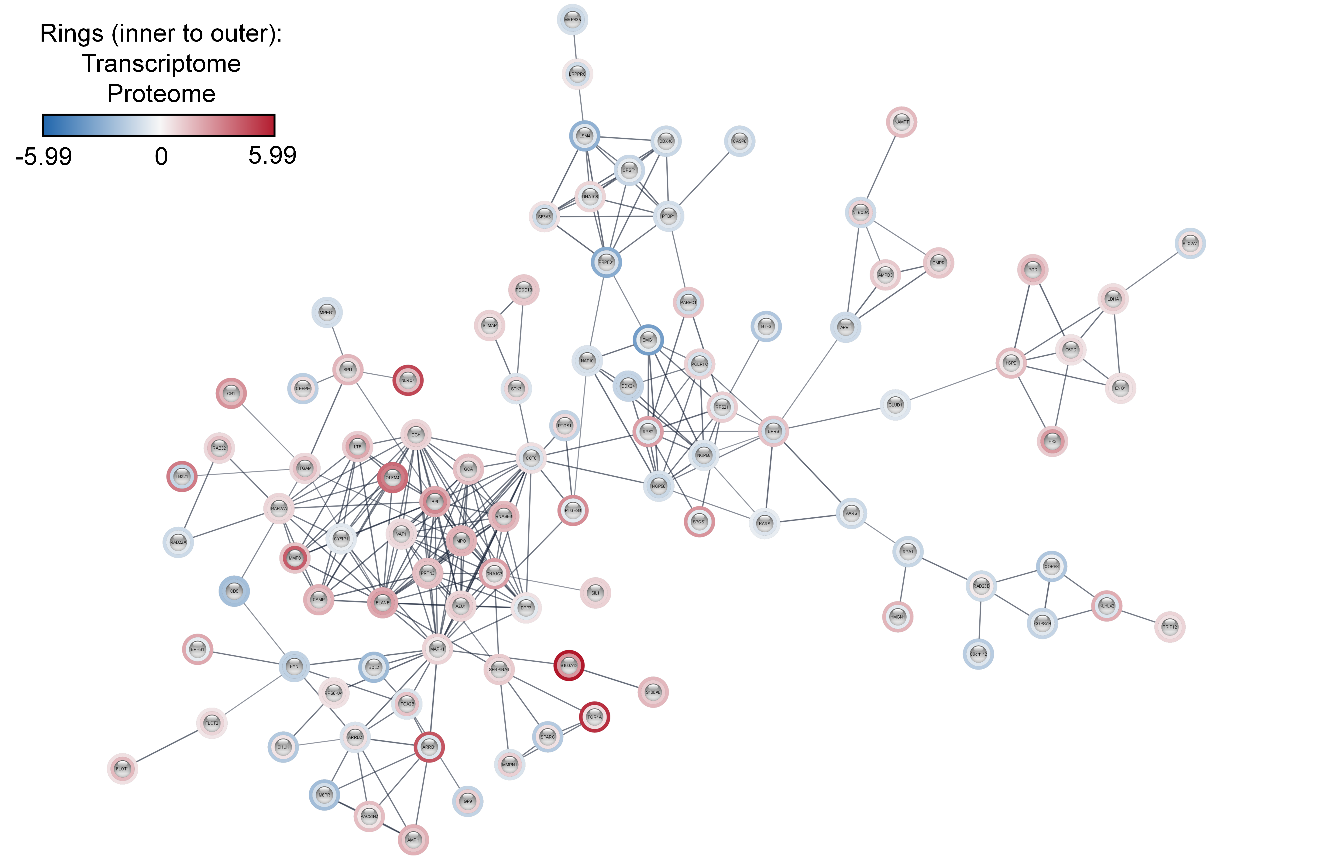


**Figure S4** - Co-differentially expressed sepsis PPIN.

**Supplementary Tables**

**Table S1.** Clinical variables and demographic data from septic patients

| ***Demographic data*** | **Sepsis (n = 24)** |
| --- | --- |
| Age, mean ± SD, year | 70.20 ± 14.41 |
| Sex (% of male) | 45.83 |
| ***Severity of disease*** |  |
| Septic shock (%) | 41.66 |
| Mortality (%) | 20.83 |
| ***Severity scores, mean ± SD*** |  |
| SOFA | 5.52 ± 3.20 |
| ***Organ dysfunction (%)*** |  |
| Renal | 29.16 |
| Coagulation | 8.33 |
| Respiratory | 33.33 |
| Hepatological | 12.5 |
| ***Chronic comorbidity (%)*** |  |
| AIDS | 0 |
| COPD | 8.33 |
| Diabetes | 29.16 |
| Chronic renal disease | 0 |
| Cardiovascular insufficiency | 4.16 |

**Table S2.** Primer sequences for the quantitative RT-PCR.

| **Gene** | **Forward Sequence** | **Reverse Sequence** |
| --- | --- | --- |
| *STX5* | AAAGCGCAAGTCCCTCTTTGA | TGAGCAATTTGTTTGTTGAGGC |
| *RPL11* | AAAGGTGCGGGAGTATGAGTT | TCCAGGCCGTAGATACCAATG |
| *EIF2AK2* | TGGAAAGCGAACAAGGAGTAAG | CCAAAGCGTAGAGGTCCACTT |
| *RPS21* | AGCAATCGCATCATCGGTG | CCCCGCAGATAGCATAAGTTTTA |
| *POLR2H* | TGACCGAGTGTCTCGACTG | GTCACCCAAGTCTACAGGGTAA |
| *NCBP2* | AAAACGCCATGCGGTACATAA | GCCTGCCCTCCTTAAAGCC |
| *GTF2F1* | AAGTACGGCATCGTCCTCAAG | TGTTCTCTGTTACGCCTCCCT |
| *MRRF* | CCTTTCGCAATTATCTTGCAGC | TGGTAGCAAAATGGCGGACTG |
| *GFM1* | GAGGAAGCAGGTTAATTGGAAGG | CCCAGAATCAATGTGAGCTGAGA |
| *MRPS34* | CTCCGGGCCATGATTATCGC | CCATGCGTATCCTCTGCACAT |
| *ADAM8* | GAGGGTGAGCTACGTCCTTG | CAGCCGTATAGGTCTCTGTGT |
| *CLEC4D* | CTGATACCTTCGGTTATTGCTGT | GCACTCCTGTGCCTCTCTTAC |
| *GCA* | TGCATTCAAAGAGCTATGGGC | CTGTGCCACTTCCATCTTGAT |
| *ELANE* | GGAGCCCATAACCTCTCGC | GAGCAAGTTTACGGGGTCGT |
